# Variation in Opioid Agonist Dosing in Clinical Trials by Race and Ethnicity

**DOI:** 10.1001/jamanetworkopen.2024.36612

**Published:** 2024-10-04

**Authors:** Rachael K. Ross, Shodai Inose, Matisyahu Shulman, Edward V. Nunes, Lauren C. Zalla, A. Kathleen Burlew, Kara E. Rudolph

**Affiliations:** 1 Department of Epidemiology, Mailman School of Public Health, Columbia University, New York, New York; 2 Department of Psychiatry, Columbia University Irving Medical Center, Columbia University, New York, New York; 3 New York State Psychiatric Institute, New York, New York; 4 Department of Epidemiology, Bloomberg School of Public Health, The Johns Hopkins University, Baltimore, Maryland; 5 Department of Psychology, University of Cincinnati, Cincinnati, Ohio

## Abstract

**Importance:**

Racial and ethnic disparities in access to treatment and quality of treatment for opioid use disorder (OUD) have been identified in usual care settings. In contrast, disparities in treatment quality within clinical trials are relatively unexamined.

**Objective:**

To estimate racial and ethnic differences in the dose of opioid agonist treatment for OUD in the first 4 weeks of treatment in clinical trials.

**Design, Setting, and Participants:**

This cohort study performed analysis of the methadone and buprenorphine treatment arms of 3 trials conducted by the National Institute on Drug Abuse Clinical Trials Network between May 2006, and January 31, 2017, at multiple Clinical Trials Network sites across the US. Trial participants who were randomized to and initiated buprenorphine or methadone treatment and who identified as Hispanic, non-Hispanic Black, or non-Hispanic White were included in the present study. Data were analyzed from November 1, 2023, to August 5, 2024.

**Exposure:**

Combined race and ethnicity as self-classified by the patient at trial enrollment.

**Main Outcomes and Measures:**

The maximum daily dose of buprenorphine or methadone received in each week for the first 4 weeks of treatment. The mean dose and the percentage of patients receiving a higher dose (buprenorphine *≥*16 mg and methadone *≥*60 mg) were compared across race and ethnicity groups.

**Results:**

A total of 1748 patients (1263 who initiated buprenorphine and 485 who initiated methadone treatment) were included in the analysis (1168 [66.8%] male; median age, 33 [IQR, 26-45] years). Of these, 138 patients (7.9%) identified as Black, 273 (15.6%) as Hispanic, and 1337 (76.5%) as White. In week 4, Black patients received buprenorphine doses 2.5 (95% CI −4.6 to −0.5) mg lower and methadone doses 16.7 (95% CI, −30.7 to −2.7) mg lower compared with White patients, after standardizing by age and sex. In week 4, the percentage of patients receiving a higher dose of medication (buprenorphine *≥*16 mg; methadone *≥*60 mg) was 16.9 (95% CI, −31.9 to −1.9) points lower for Black patients compared with White patients. Hispanic and White patients received similar buprenorphine doses; Hispanic patients received lower methadone doses than White patients.

**Conclusions and Relevance:**

In this cohort study of data from 3 clinical trials, White patients generally received higher doses of medication than Black patients. Future research is needed to understand the mechanisms of and interventions to reduce disparities in OUD treatment quality and how such disparities impact generalizability of trial results.

## Key Points

**Question:**

Are there racial and ethnic disparities in buprenorphine or methadone dose for the treatment of opioid use disorder (OUD) in clinical trials?

**Findings:**

In this cohort study of 1748 participants, the mean dose and the percentage of patients receiving a higher dose were lower for non-Hispanic Black patients than for non-Hispanic White patients. Mean dose and percentage of patients receiving a higher dose were similar between Hispanic and non-Hispanic White patients.

**Meaning:**

These findings suggest the need for research to understand mechanisms associated with disparities in OUD treatment quality and interventions to reduce them.

## Teaser

This cohort study estimates disparities in doses of opioid agonist medication to treat opioid use disorder among Black, Hispanic, and White patients in 3 clinical trials.

## Introduction

Methadone and buprenorphine are the standard treatment for opioid use disorder (OUD).^1^ The effectiveness of the treatment depends on dose, with higher doses generally being more effective.^2,3,4^ Treatment Improvement Protocol 63^5^ recommends a target dose of 16 mg, with 24 mg as the upper limit, for buprenorphine and between 80 and 120 mg for methadone, though higher doses may be needed with the increased prevalence of fentanyl and other synthetic opioids.^6^

There are persistent racial and ethnic disparities in access to and quality of treatment for OUD.^7^ Compared with Black and Hispanic or Latinx patients, White patients are more likely to be prescribed medication and to receive higher doses of medication.^8,9^ These disparities have been identified in usual care settings, outside experimental research. It is unknown whether disparities persist in experimental settings, such as comparative effectiveness trials that randomize medication but not dose. Examining treatment disparities in experimental settings is important for understanding the generalizability of clinical trial evidence that informs treatment recommendations. We estimated disparities in dose in 3 clinical trials conducted in the National Institute on Drug Abuse Clinical Trials Network.

## Methods

### Data Sources

We analyzed harmonized data from the Starting Treatment With Agonist Replacement Therapies trial (START),^10^ phase 2 of the Prescription Opioid Addiction Treatment Study (POATS),^11^ and the Extended-Release Naltrexone vs Buprenorphine for Opioid Treatment trial (X:BOT).^12^ These data have been described previously.^13,14^ These trials were conducted between May 2006 and January 31, 2017 (START: May 2006 to August 2010; phase 2 of POATS: June 12, 2006, to July 9, 2009; and X:BOT: January 30, 2014, to January 31, 2017), a period that included time before and during the early emergence of fentanyl into the US opioid supply. The analysis for the present study was conducted from November 1, 2023, to August 5, 2024.

We included patients who were randomized to and initiated methadone (START) or buprenorphine (all 3 trials) treatment. We restricted analysis to the first 4 weeks of treatment, which is the primary period of dose adjustment. The Institutional Review Board at the New York State Psychiatric Institute determined this analysis of deidentified data to be nonhuman participant research and did not require informed consent. This study follows the Strengthening the Reporting of Observational Studies in Epidemiology (STROBE) reporting guideline.

### Definitions

At trial enrollment, participants self-classified their race and ethnicity at the time of trial enrollment, using fixed categories that differed by trial; responses were harmonized previously,^13^ and additional details are provided in the eText in Supplement 1.^15^ We restricted the analysis to patients who identified as Hispanic or Latinx, non-Hispanic or non-Latinx Black, and non-Hispanic or non-Latinx White (hereafter referred to as Hispanic, Black, and White, respectively) due to sample size. We conceptualize race and ethnicity as social constructs.

For each of the first 4 weeks of treatment, we assessed the maximum daily dose for each patient. Buprenorphine was dispensed weekly in POATS and X:BOT and either daily or 3 times weekly in START. Study protocols encouraged rapid induction (1-3 days) with a maximum dose of 32 mg (doses >24 mg required prior approval in POATS). In START, methadone was dispensed daily with no explicit maximum. Protocols encouraged dose adjustments in response to opioid use, craving, or withdrawal. In data cleaning, we carried forward the last documented daily dose until there was a dose change, medication treatment was discontinued, or the patient experienced a relapse (7 consecutive days of nonstudy opioid use starting 14 days after enrollment).^14^ Covariates measured at enrollment included sex, age, severity of withdrawal symptoms (based on Clinical Opiate Withdrawal Scale or Subjective Opiate Withdrawal Scale), other substance use disorders (eg, alcohol use disorder, cocaine use disorder), comorbid conditions (reported history of bipolar disorder, anxiety or panic disorder, or major depressive disorder), and drug use in the 30 days before randomization (reported use of amphetamines, cannabis, benzodiazepines, or intravenous drugs).

### Statistical Analysis

We aimed to assess racial and ethnic disparities in opioid agonist treatment dose. For each week, we estimated difference in mean (maximum daily) dose among racial and ethnic groups. In weeks 3 and 4, we also dichotomized dose as higher (buprenorphine ≥16 mg and methadone ≥60 mg) or lower^3^ and compared the percentage of patients receiving a higher dose between groups. Racial and ethnic disparities in dose in these data may reflect (1) differences that were present before trial enrollment, including differential access to specific trials, and (2) differences in treatment decisions in the trial. We implemented analyses that attempted to capture both sources of disparity.

Adjustment for covariates removes differences in their distribution among racial and ethnic groups (ie, the covariate distribution in each racial and ethnic group is standardized to a common distribution). Thus, the choice of which covariates to adjust for reflects a value judgment: a distinction between “allowable” and “nonallowable” sources of difference among groups.^16,17^ Briefly, a researcher may deem some sources of difference allowable, that is, not unfair or unjust, and remove their contribution to a disparity through adjustment. In contrast, other sources of difference deemed nonallowable are considered unjust or unfair and are not adjusted away (ie, the estimated disparity encompasses pathways through these sources). We considered 2 adjustment sets, varying the covariates considered allowable. First, we conducted a minimally adjusted analysis, standardizing by age and sex. Here we considered age and sex as allowable differences, but we considered differences in other covariates such as drug use history, comorbidities, and withdrawal symptom severity as nonallowable. This analysis estimated an overall disparity in dose, encompassing differences that were present at enrollment, which may reflect population-level disparities in drug use and comorbidity burden or disparities in the trial recruitment process, as well as differences in treatment in the trial. Second, we conducted an analysis that adjusted for all the measured covariates, which may be reasonably used by trial clinicians to inform dose. This second analysis examined disparities in treatment decisions made by clinicians, which should be based on how patients present to care. Comparison of the direction and magnitude of disparities between these analyses allows us to assess whether disparities persist after removing differences in measured covariates at presentation. Given the sample sizes and the number of covariates (Table 1), precision is poor in the analysis adjusting for all covariates (ie, 95% CIs are wide). Per recommendations in the fields of statistics and clinical research, we do not base our conclusions on statistical significance testing; instead, we interpret the magnitude, direction, and precision of estimates.^18,19,20,21^

**Table 1.** Description of Patient Sample Included in Analysis

Baseline covariates	Treatment group, patients, No. (%)^a^					
	Buprenorphine (n = 1263)			Methadone (n = 485)		
	Hispanic (n = 193)	Non-Hispanic Black (n = 92)	Non-Hispanic White (n = 978)	Hispanic (n = 80)	Non-Hispanic Black (n = 46)	Non-Hispanic White (n = 359)
Sex						
Male	134 (69.4)	68 (73.9)	639 (65.3)	60 (75.0)	34 (73.9)	233 (64.9)
Female	59 (30.6)	24 (26.1)	339 (34.7)	20 (25.0)	12 (26.1)	126 (35.1)
Age, median (IQR), y	37 (28-46)	46 (36-52)	31 (26-41)	41 (31-48)	50 (46-55)	33 (26-43)
Moderate, moderately severe, or severe withdrawal^b^	83 (43.5)	27 (29.3)	523 (53.7)	39 (48.8)	17 (37.0)	160 (44.9)
No. missing	2	0	4	0	0	3
Alcohol use disorder^c^	47 (25.3)	13 (14.3)	190 (19.5)	9 (11.7)	7 (15.9)	88 (25.1)
No. missing	7	1	6	3	2	8
Cocaine use disorder^c^	49 (26.3)	30 (33.0)	234 (24.1)	17 (22.1)	22 (50.0)	120 (34.2)
No. missing	7	1	8	3	2	8
History of bipolar disorder	22 (11.4)	5 (5.4)	108 (11.1)	5 (6.3)	2 (4.3)	50 (14.0)
No. missing	0	0	1	0	0	1
History of anxiety disorder	64 (33.2)	14 (15.2)	344 (35.2)	18 (22.5)	7 (15.2)	128 (35.8)
No. missing	0	0	0	0	0	1
History of major depression	62 (32.1)	16 (17.4)	308 (31.5)	15 (18.8)	9 (19.6)	108 (30.1)
Baseline amphetamine use^d^	38 (19.7)	4 (4.3)	121 (12.4)	12 (15.0)	3 (6.5)	47 (13.1)
No. missing	0	0	1	0	0	1
Baseline cannabis use^d^	66 (34.2)	24 (26.1)	360 (36.8)	14 (17.5)	4 (8.7)	116 (32.4)
No. missing	0	0	1	0	0	1
Baseline benzodiazepine use^d^	34 (17.6)	15 (16.3)	253 (25.9)	9 (11.3)	3 (6.5)	67 (18.7)
No. missing	0	0	1	0	0	1
Baseline IV drug use^d^	122 (67.8)	44 (49.4)	461 (48.2)	61 (76.3)	31 (67.4)	247 (69.0)
No. missing	13	3	22	0	0	1

Abbreviation: IV, intravenous.

^a^ Race and ethnicity were self-classified, with response options differing by trial. More details are provided in the eText in Supplement 1.

^b^ Based on highest Clinical Opiate Withdrawal Scale (COWS) score or Subjective Opiate Withdrawal Scale (SOWS) score at or before randomization; moderate, moderately severe, or severe withdrawal were characterized as scores above 13 for COWS and above 11 for SOWS.

^c^ Indicates past year.

^d^ Indicates use 30 days before randomization.

For the analysis of buprenorphine, we combined data from 3 trials (the methadone data were obtained from only 1 trial). To examine variation by trial, we implemented 2 additional analyses standardized by age and sex: (1) stratified by trial and (2) including an indicator for trial in the adjustment set. POATS had a large sample size (n = 345) but included only 8 Black patients and 18 Hispanic patients (eTable 1 in Supplement 1). Given these small numbers, the latter analysis including an indicator for trial was conducted with and without POATS.

Finally, age- and sex-standardized analyses for both buprenorphine and methadone treatment were repeated stratified by severity of baseline withdrawal symptoms (moderate or severe and mild or none) categorized from the Clinical Opiate Withdrawal Scale or Subjective Opiate Withdrawal Scale. This analysis was conducted after observing symptom severity differences by race and ethnicity.

To implement standardization and to account for censoring due to loss to follow-up or relapse, we used a targeted maximum likelihood estimator.^22^ This estimator relies on estimating a model for race and ethnicity that is conditional on covariates; a model for censoring that is conditional on race and ethnicity, covariates, and prior dose; and a model for dose that is conditional on race and ethnicity and covariates. These models were fit using the SuperLearner ensemble machine learning algorithm with the following candidate learners: intercept-only mean models, main-effects generalized linear models, regularized regression models, multivariate adaptive regression splines, random forests, and eXtreme Gradient Boosting.^23^ We estimated the variance using the sample variance of the efficient influence function and constructed Wald-type 95% CIs. Covariate missing values were imputed with the mode, stratified by treatment. See eText in Supplement 1 for additional analysis details. Code for analysis is available on GitHub.

## Results

### Cohort

Our analysis included 1748 patients; 138 (7.9%) identified as Black, 273 (15.6%) identified as Hispanic, and 1337 (76.5%) as White (Table 1). There were 1263 patients who initiated buprenorphine treatment (665 from START, 345 from POATS, and 253 from X:BOT) and 485 patients who initiated methadone (eTable 1 and eFigure 1 in Supplement 1). The sample was mostly male (1168 [66.8%] vs 580 [33.2%] female), with a median age of 33 (IQR, 26-45) years. Black patients had the highest median age, while White patients had the lowest. White and Hispanic patients had a higher reported history of psychiatric conditions at baseline than Black patients. A greater portion of White and Hispanic patients reported moderate or severe withdrawal symptoms at baseline compared with Black patients.

### Continuous Dose

#### Buprenorphine

The maximum daily buprenorphine dose ranged from 2 to 32 mg (median, 16 [IQR, 12-24] mg). Figure, A, shows the distribution of buprenorphine doses received by patients in each racial and ethnic group, by week. In week 1, Black patients received a mean of 15.5 (6.5) mg; Hispanic patients, 16.7 (7.4) mg; and White patients, 17.0 (7.2) mg. In week 4, Black patients received a mean of 17.7 (7.1) mg; Hispanic patients, 19.0 (7.7) mg; and White patients, 19.3 (7.3) mg.

**Figure.**
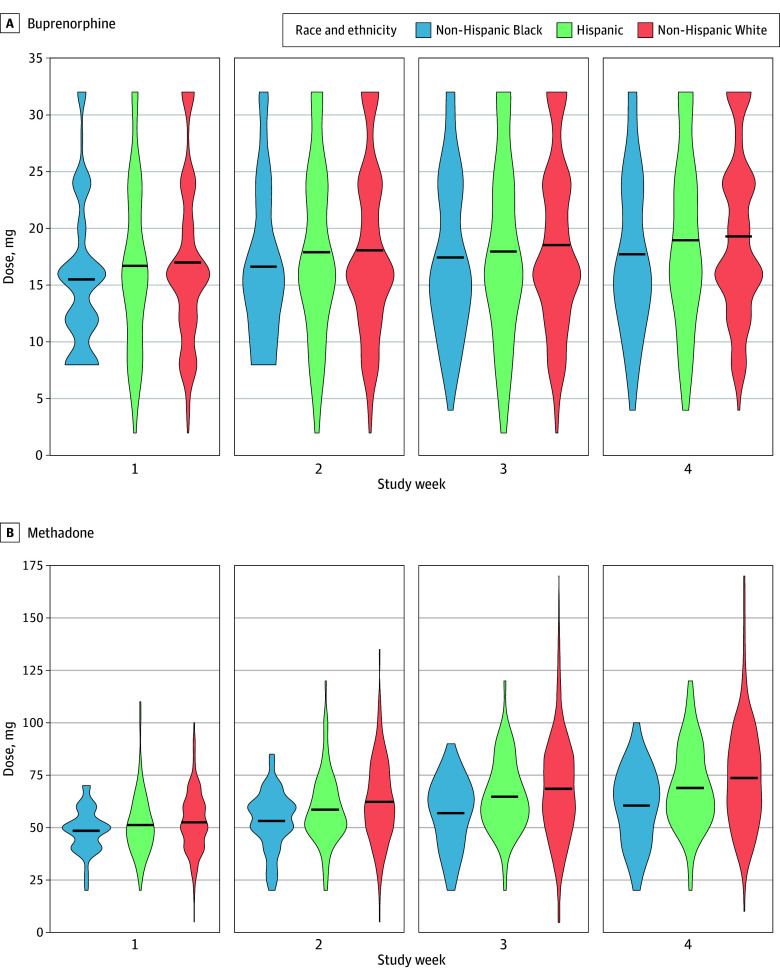
Violin Plots of Maximum Daily Dose Opioid Agonist Treatment in the First 4 Weeks of Treatment Data are stratified by self-classified race and ethnicity. The black horizontal line indicates the mean.

Black patients received lower mean doses than White patients in all weeks (Table 2). For example, in week 4 (the week with the largest difference), standardized by age and sex, Black patients were prescribed doses 2.5 (95% CI, −4.6 to −0.5) mg lower than White patients. The magnitude of differences was similar when standardizing by all covariates, though precision decreased (ie, 95% CIs were wider). For example, the week 4 difference between Black and White patients was −2.3 (95% CI, −6.2 to 1.6) mg.

**Table 2.** Maximum Daily Dose of Buprenorphine by Week of Treatment Stratified by Race and Ethnicity

Study week	Patient group^a^				
	Hispanic		Non-Hispanic Black		Non-Hispanic White, mean dose (95% CI), mg
	Mean dose (95% CI), mg	Difference (95% CI), mg^b^	Mean dose (95% CI), mg	Difference (95% CI), mg^b^	
**Standardized by age and sex**					
1	16.6 (15.5 to 17.7)	−0.4 (−1.7 to 0.8)	15.2 (13.2 to 17.2)	−1.8 (−3.9 to 0.2)	17.0 (16.6 to 17.5)
2	17.8 (16.7 to 18.9)	−0.3 (−1.6 to 0.9)	15.9 (14.0 to 17.8)	−2.2 (−4.2 to −0.3)	18.1 (17.6 to 18.6)
3	17.8 (16.6 to 19.0)	−0.8 (−2.1 to 0.5)	16.2 (14.3 to 18.1)	−2.4 (−4.4 to −0.4)	18.6 (18.1 to 19.1)
4	18.9 (17.6 to 20.2)	−0.5 (−1.9 to 0.9)	16.8 (14.8 to 18.8)	−2.5 (−4.6 to −0.5)	19.4 (18.8 to 19.9)
**Standardized by full covariates set**					
1	16.9 (15.7 to 18.1)	−0.1 (−1.3 to 1.2)	15.1 (12.4 to 17.7)	−1.9 (−4.6 to 0.8)	17.0 (16.5 to 17.4)
2	18.0 (16.7 to 19.2)	0.0 (−1.4 to 1.3)	15.8 (13.2 to 18.3)	−2.2 (−4.9 to 0.4)	18.0 (17.5 to 18.5)
3	17.8 (16.5 to 19.2)	−0.6 (−2.0 to 0.8)	16.7 (14.0 to 19.4)	−1.8 (−4.5 to 1.0)	18.5 (18.0 to 19.0)
4	19.1 (17.8 to 20.5)	−0.2 (−1.7 to 1.2)	17.0 (13.2 to 20.9)	−2.3 (−6.2 to 1.6)	19.3 (18.8 to 19.9)

^a^ Race and ethnicity were self-classified, with response options differing by trial. More details are provided in the eText in Supplement 1.

^b^ Difference compared with non-Hispanic White patients.

Hispanic patients received mean doses similar to or just slightly lower than White patients (Figure, A, and Table 2). Standardized by age and sex, weekly differences between Hispanic and White patients varied from −0.3 (95% CI, −1.6 to 0.9) mg (in week 2) to −0.8 (95% CI, −2.1 to 0.5) mg (in week 3). With standardization by all covariates, differences between Hispanic and White patients were attenuated and close to zero, except in week 3.

The data on buprenorphine dose were pooled from all 3 clinical trials. eFigure 2 in Supplement 1 shows the distribution of buprenorphine doses stratified by trial. eTable 2 in Supplement 1 presents week 4 results from analyses stratified by trial and analyses adjusting for trial. We found that differences between Black and White patients in week 4 varied by trial. In POATS, the difference was positive (0.9 [95% CI, −6.9 to 8.7] mg). The START trial had the largest difference (−2.9 [95% CI, −5.3 to −0.5] mg). Compared with analyses ignoring trial, the difference between Black and White patients at week 4 in analyses adjusting for trial were similar when POATS was excluded (−2.5 [95% CI, −4.3 to −0.6] mg) and smaller when POATS was included (−1.6 [95% CI, −3.7 to 0.5] mg).

In contrast to the other trials, Hispanic patients received higher doses than White patients in POATS (3.7 [95% CI, −0.2 to 7.6] mg). Consequently, analyses adjusting for trial found no dose difference between Hispanic and White patients when POATS was included (0.1 [95% CI, −1.5 to 1.7] mg) and a negative dose difference when POATS was excluded (−1.4 [95% CI, −2.9 to 0.1] mg).

#### Methadone

The maximum daily methadone dose ranged from 5 to 170 mg (median, 60 [IQR, 50-75] mg). The Figure, B, shows the distribution of methadone doses received by patients in each racial and ethnic group, by week. In week 1, Black patients received a mean of 48.7 (10.6) mg; Hispanic patients, 51.2 (14.7) mg; and White patients, 52.7 (14.3) mg. In week 4, Black patients received a mean of 60.6 (18.6) mg; Hispanic patients, 69.0 (19.9) mg; and White patients, 73.9 (25.4) mg.

Black patients received mean lower doses than White patients in all weeks (Table 3). The size of the dose difference increased each week, with the largest difference observed in week 4 (−16.7 [95% CI, −30.7 to −2.7] mg). The difference in dose between Black and White patients remained although slightly smaller, after standardizing by all covariates. For example, the week 4 difference was −13.8 (95% CI, −25.3 to −2.4) mg.

**Table 3.** Maximum Daily Dose of Methadone by Week of Treatment Stratified by Race and Ethnicity

Study week	Patient group^a^				
	Hispanic		Non-Hispanic Black		Non-Hispanic White, mean dose (95% CI), mg
	Mean dose (95% CI), mg	Difference (95% CI), mg^b^	Mean dose (95% CI), mg	Difference (95% CI), mg^b^	
**Standardized by age and sex**					
1	50.8 (47.3 to 54.3)	−1.8 (−5.6 to 2.0)	45.0 (37.2 to 52.7)	−7.7 (−15.6 to 0.2)	52.6 (51.1 to 54.1)
2	58.7 (54.1 to 63.2)	−3.5 (−8.4 to 1.5)	49.6 (40.7 to 58.5)	−12.5 (−21.6 to −3.4)	62.1 (60.2 to 64.1)
3	64.9 (59.5 to 70.3)	−3.4 (−9.4 to 2.6)	52.6 (42.1 to 63.1)	−15.7 (−26.5 to −4.8)	68.3 (65.7 to 70.9)
4	68.6 (62.5 to 74.8)	−4.6 (−11.3 to 2.2)	56.5 (42.8 to 70.2)	−16.7 (−30.7 to −2.7)	73.2 (70.4 to 76.1)
**Standardized by full covariates set**					
1	50.9 (47.0 to 54.8)	−1.8 (−5.9 to 2.4)	46.7 (38.8 to 54.7)	−6.0 (−14.0 to 2.1)	52.7 (51.2 to 54.2)
2	59.2 (54.0 to 64.3)	−2.9 (−8.4 to 2.7)	51.6 (40.0 to 63.2)	−10.4 (−22.2 to 1.4)	62.0 (60.0 to 64.0)
3	63.9 (56.5 to 71.2)	−4.3 (−12.1 to 3.6)	55.3 (41.4 to 69.1)	−12.9 (−27.0 to 1.2)	68.1 (65.5 to 70.7)
4	68.6 (61.7 to 75.5)	−4.3 (−11.8 to 3.2)	59.0 (48.0 to 70.1)	−13.8 (−25.3 to −2.4)	72.9 (70.0 to 75.8)

^a^ Race and ethnicity were self-classified, with response options differing by trial. More details are provided in the eText in Supplement 1.

^b^ Difference compared with non-Hispanic White patients.

Hispanic patients also received lower doses than White patients, though differences (eg, −4.6 [95% CI, −11.3 to 2.2] mg in week 4) were smaller than those between Black and White patients. Differences between Hispanic and White patients remained similar in most weeks after standardizing for all covariates (eg, −4.3 [95% CI, −11.8 to 3.2] mg in week 4).

We identified 6 outlier methadone doses in week 3 and 5 outlier doses in week 4 (identified as more extreme than the upper limits of the Tukey fence). All outlier doses were received by White patients. We conducted a sensitivity analysis replacing these outliers with the highest observed nonoutlier dose in each week (120 mg in week 3 and 140 mg in week 4). Results after modifying these outliers were similar to those of the primary analysis results (eTable 3 in Supplement 1).

### Higher or Lower Dose

Most patients received a dose of at least 16 mg for buprenorphine (731 [73.0%]) or at least 60 mg for methadone (273 [70.0%]) in both weeks 3 and 4. Standardized by age and sex, the percentage of patients receiving a higher dose was lower for Black patients than White patients in both weeks (Table 4): 19.9 (95% CI, −34.4 to −5.4) percentage points lower in week 3 and 16.9 (95% CI, −31.9 to −1.9) percentage points lower in week 4. When standardizing by all covariates, differences between Black and White patients were smaller, though the data were still most compatible with a lower percentage of Black patients receiving a higher dose than the percentage of White patients (week 3: −11.5 [95% CI, −26.7 to 3.8] percentage points lower; week 4: −10.6 [95% CI, −28.8 to 7.6] percentage points lower). The percentage of patients receiving a higher dose was similar among Hispanic and White patients (eg, week 3: −2.1 [95% CI, −9.2 to 5.1] percentage points lower) and did not differ by the covariate standardization set.

**Table 4.** Percentage of Patients Receiving a Higher Dose of Buprenorphine or Methadone by Week of Treatment Stratified by Race and Ethnicity^a^

Study week	Patient group^b^				
	Hispanic		Non-Hispanic Black		Non-Hispanic White, % (95% CI)
	% (95% CI)	Difference, percentage points (95% CI)^c^	% (95% CI)	Difference, percentage points (95% CI)^c^	
**Standardized by age and sex**					
3	68.9 (62.6 to 75.2)	−2.6 (−9.4 to 4.2)	51.6 (37.3 to 65.9)	−19.9 (−34.4 to −5.4)	71.5 (68.9 to 74.1)
4	73.0 (66.6 to 79.3)	−2.0 (−8.9 to 4.9)	58.1 (43.3 to 72.8)	−16.9 (−31.9 to −1.9)	75.0 (72.3 to 77.6)
**Standardized by full covariates set**					
3	68.9 (62.3 to 75.5)	−2.1 (−9.2 to 5.1)	59.5 (44.5 to 74.6)	−11.5 (−26.7 to 3.8)	71.0 (68.4 to 73.6)
4	72.8 (65.8 to 79.7)	−1.9 (−9.3 to 5.6)	64.0 (46.0 to 82.0)	−10.6 (−28.8 to 7.6)	74.6 (71.9 to 77.3)

^a^ Indicates 16 mg or more of buprenorphine and 60 mg or more of methadone.

^b^ Race and ethnicity were self-classified, with response options differing by trial. More details are provided in the eText in Supplement 1.

^c^ Difference compared with non-Hispanic White patients.

### Stratification by Withdrawal Symptom Severity

eTable 4 in Supplement 1 presents results from analyses stratified by severity of withdrawal symptoms reported at baseline. This analysis was conducted after observing that severity of documented withdrawal symptoms at baseline was lower for Black patients than White patients. Differences in doses received by Black and White patients were present in both strata and both medications, though the magnitude of the differences varied; for example, the methadone dose difference was larger among patients who reported mild or no symptoms (−14.4 [−27.3 to −1.4] mg) than among patients who reported moderate or severe symptoms (−10.4 [−18.7 to −2.2] mg), though 95% CIs were wide. The results comparing doses received by Hispanic and White patients stratified by withdrawal severity varied by stratum and medication (eTable 4 in Supplement 1).

## Discussion

In this cohort study, we observed that Black patients received lower buprenorphine and methadone doses than White patients in the initial weeks of treatment in clinical trials, consistent with prior research examining methadone dosing.^8,9^ Disparities in dosing in performing our analysis persisted despite standardized study protocols with treatment guidance and research-informed clinicians. Importantly, higher doses are associated with better outcomes^2,3,4^; thus, disparities in dose may contribute to disparities in treatment outcomes. Results generally supported Hispanic patients receiving doses similar to White patients, though there was variation in results across analyses.

To estimate disparities, we conducted 2 main analyses, varying the adjustment set. Although precision varied across analyses (as expected given the sample size and number of covariates), the direction and magnitude of disparities between Black and White patients were generally robust to adjustment, implying that these measured individual-level covariates did not fully explain the observed differences. While disparities in dosing in usual care settings outside of research are a substantial treatment concern, disparities in experimental settings raise additional issues. Specifically, if certain groups are subject to inadequate dosing in such settings, the applicability of the research findings to those groups is compromised. Although identifying the factors that explain these disparities is beyond the scope of this report, others^24,25,26^ have speculated that disparities in dose may be the results of multiple intertwined factors rooted in structural, institutional, and interpersonal racism. Some investigators^9^ suggest disparities stem from structural factors related to facility characteristics and residential segregation. Others^7,27,28^ speculate that clinician characteristics such as conscious or unconscious racist beliefs may underlie differential dosing. Other studies^26,29,30,31^ have suggested patient factors such as lack of trust in the health care system as a result of historical discrimination and ongoing racist practices may also play a role. In our study, disparities likely result from many factors. For example, we observed that documented severity of baseline withdrawal symptoms differed between Black and White patients. This baseline difference could reflect fewer withdrawal symptoms among Black patients in these trials, possibly because of the enrollment process (eg, timing) at facilities that enrolled more Black patients. It is also possible that this baseline difference reflects differences in how symptom scores were elicited, documented, or reported based on a patient’s race. We are not able to discern the causes of baseline differences in withdrawal symptoms in our data source. Because guidelines recommend dose changes based on withdrawal symptom severity,^5^ this example illustrates how disparities in dosing could be perpetuated even when clinicians follow treatment recommendations.

In response to disparities in treatment access and quality outside of the research context, a variety of remedies have been proposed, including campaigns to improve awareness of dosing recommendations,^9^ training for facility staff and managers on how to mitigate the impact of interpersonal racism on clinical decision-making,^8,9^ oversight by accrediting bodies to assess treatment quality at treatment facilities,^9^ and community-specific interventions that aim to address mistrust of safety and effectiveness of OUD treatment.^25,32^

### Limitations

This study has some limitations. The data analyzed herein were collected in trials, conducted over a 10-year period that ended more than 8 years ago. This study period includes time before and in the early years of the emergence of fentanyl. For methadone, all data predate the emergence of fentanyl. Therefore, the results may not reflect dosing patterns present in more recent trials, and the importance of higher doses due to fentanyl was not a concern for clinicians in these trials. Our sample size, particularly of non-Hispanic Black patients, prevented us from examining whether disparities are driven by differences in dosing between treatment facilities that treat patient populations with differing distributions of race and ethnicity, as has been seen in usual care settings.^9^ Furthermore, we did not have the data to investigate mechanisms for dosing disparities (eg, extent to which dosing differences are driven by patient preference for lower doses) or to explore potential remedies.

## Conclusions

In this cohort study, we found evidence of racial disparities in OUD medication dosing in clinical trials, similar to those found in usual care settings. Future research is critical to understand driving mechanisms of racial and ethnic disparities in quality of treatment for OUD and interventions to reduce these.

## Notes

Supplement 1. **eText.** Characterization of Participant Race and Ethnicity and Details of Analysis **eFigure 1.** Sample Size by Week **eFigure 2.** Violin Plots of Maximum Daily Dose of Buprenorphine Stratified by Self-Classified Race and Ethnicity for Each Trial **eTable 1.** Description of Patient Sample Included in Buprenorphine Analysis, Stratified by Trial **eTable 2.** Analyses Stratifying or Standardizing by Trial: Maximum Daily Dose of Buprenorphine in Week 4 of Treatment, Standardized by Age and Sex **eTable 3.** Mean Methadone Dose From Sensitivity Analysis in Which Methadone Outlier Doses Were Replaced, Standardized by Age and Sex **eTable 4.** Analysis Stratified by Severity of Baseline Withdrawal Symptoms: Differences in Maximum Daily Dose and Percentage Receiving a Higher Dose in Week 4 of Treatment, Standardized by Age and Sex
eReferences

Supplement 2.
Data Sharing Statement
