# Supplementary material for: Variation in Opioid Agonist Dosing in Clinical Trials by Race and Ethnicity
Source: JAMA Netw Open. 2024 Oct 4;7(10):e2436612. doi: 10.1001/jamanetworkopen.2024.36612 (PMC11581645; doi:10.1001/jamanetworkopen.2024.36612)
Supplement: Supplement 1. — eText. Characterization of Participant Race and Ethnicity and Details of Analysis eFigure 1. Sample Size by Week eFigure 2. Violin Plots of Maximum Daily Dose of Buprenorphine Stratified by Self-Classified Race and Ethnicity for Each Trial eTable 1. Description of Patient Sample Included in Buprenorphine Analysis, Stratified by Trial eTable 2. Analyses Stratifying or Standardizing by Trial: Maximum Daily Dose of Buprenorphine in Week 4 of Treatment, Standardized by Age and Sex eTable 3. Mean Methadone Dose From Sensitivity Analysis in Which Methadone Outlier Doses Were Replaced, Standardized by Age and Sex eTable 4. Analysis Stratified by Severity of Baseline Withdrawal Symptoms: Differences in Maximum Daily Dose and Percentage Receiving a Higher Dose in Week 4 of Treatment, Standardized by Age and Sex eReferences [file jamanetwopen-e2436612-s001.pdf]

Supplementary Online Content

Ross RK, Inose S, Shulman M, et al. Variation in opioid agonist dosing in clinical trials by race and ethnicity. *JAMA Netw Open*. 2024;7(10):e2436612. doi:10.1001/jamanetworkopen.2024.36612

eText. Characterization of Participant Race and Ethnicity and Details of Analysis
eFigure 1. Sample Size by Week
eFigure 2. Violin Plots of Maximum Daily Dose of Buprenorphine Stratified by Self-Classified Race and Ethnicity for Each Trial
eTable 1. Description of Patient Sample Included in Buprenorphine Analysis, Stratified by Trial
eTable 2. Analyses Stratifying or Standardizing by Trial: Maximum Daily Dose of Buprenorphine in Week 4 of Treatment, Standardized by Age and Sex
eTable 3. Mean Methadone Dose From Sensitivity Analysis in Which Methadone Outlier Doses Were Replaced, Standardized by Age and Sex
eTable 4. Analysis Stratified by Severity of Baseline Withdrawal Symptoms: Differences in Maximum Daily Dose and Percentage Receiving a Higher Dose in Week 4 of Treatment, Standardized by Age and Sex
eReferences.

This supplementary material has been provided by the authors to give readers additional information about their work.

© 2024 Ross RK et al. *JAMA Network Open*.

eText. Characterization of Participant Race and Ethnicity and Details of Analysis

Characterization of participant race and ethnicity

Patients' race and ethnicity were collected at enrollment in each trial, though the available categories varied by trial. These data were harmonized and categorized under CTN-0094 (https://github.com/CTN-0094/public.ctn0094data).

Hispanic/Latinx self-classification

In START and POATS, patients were asked to select either "Spanish origin, Hispanic or Latino" or "Not of Spanish origin, Hispanic or Latino". In X-BOT, patients were asked to select "No", "Yes", "Don't know", "Refused" to the question "Does the participant consider him or herself to be Hispanic/Latino?" Patients responding in the affirmative were classified as Hispanic/Latinx.

Race self-classification

In START and POATS, patients were asked to select all that apply from 8 choices: American Indian or Alaska Native, Asian, Black or African American, Native Hawaiian or Pacific Islander, White, Other, Participant chooses not to answer, or Unknown. In X-BOT, patients were asked to select all that apply from 18 choices: White, Black/African American, Indian (American), Alaska Native, Native Hawaiian, Guamanian, Samoan, Other Pacific Islander, Asian Indian, Chinese, Filipino, Japanese, Korean, Vietnamese, Other Asian, Some other race, Don't know, Refused. Race was ultimately categorized as White, Black, Refused/Missing, and Other as these are the predominant racial categories currently surveyed by the United States census.

Details of analysis

To implement standardization, we used the doubly robust targeted maximum likelihood estimator (TMLE) via the *lmtp* R package. [1–3] Models were fit using the Super Learner stacked ensemble learning algorithm [4] via the *SuperLearner* R package [5], consisting of the following candidate learners (with 10-fold cross-validation): intercept-only mean models; main-effects generalized linear models; regularized regression models (LASSO [6]); multivariate adaptive regression splines (MARS [7]); random forests [8]; eXtreme Gradient Boosting (XGBoost [9]). These learners were selected to provide a diverse list of methods encompassing techniques from different algorithm families. To fit each machine learning algorithm, we used 10-fold cross-validation to avoid over-fitting the data. [10] We cross-fit the TMLE with 10-folds. We estimated the variance using the sample variance of the efficient influence function and constructed Wald-type 95% confidence intervals. Covariate missing values were imputed with the mode, stratified by treatment. We included missing indicators for variables with enough variation in the missing indicator variables among folds of the cross-validation. We used R (version 4.3.1).

© 2024 Ross RK et al. *JAMA Network Open*.

eFigures

eFigure 1. Sample Size by Week

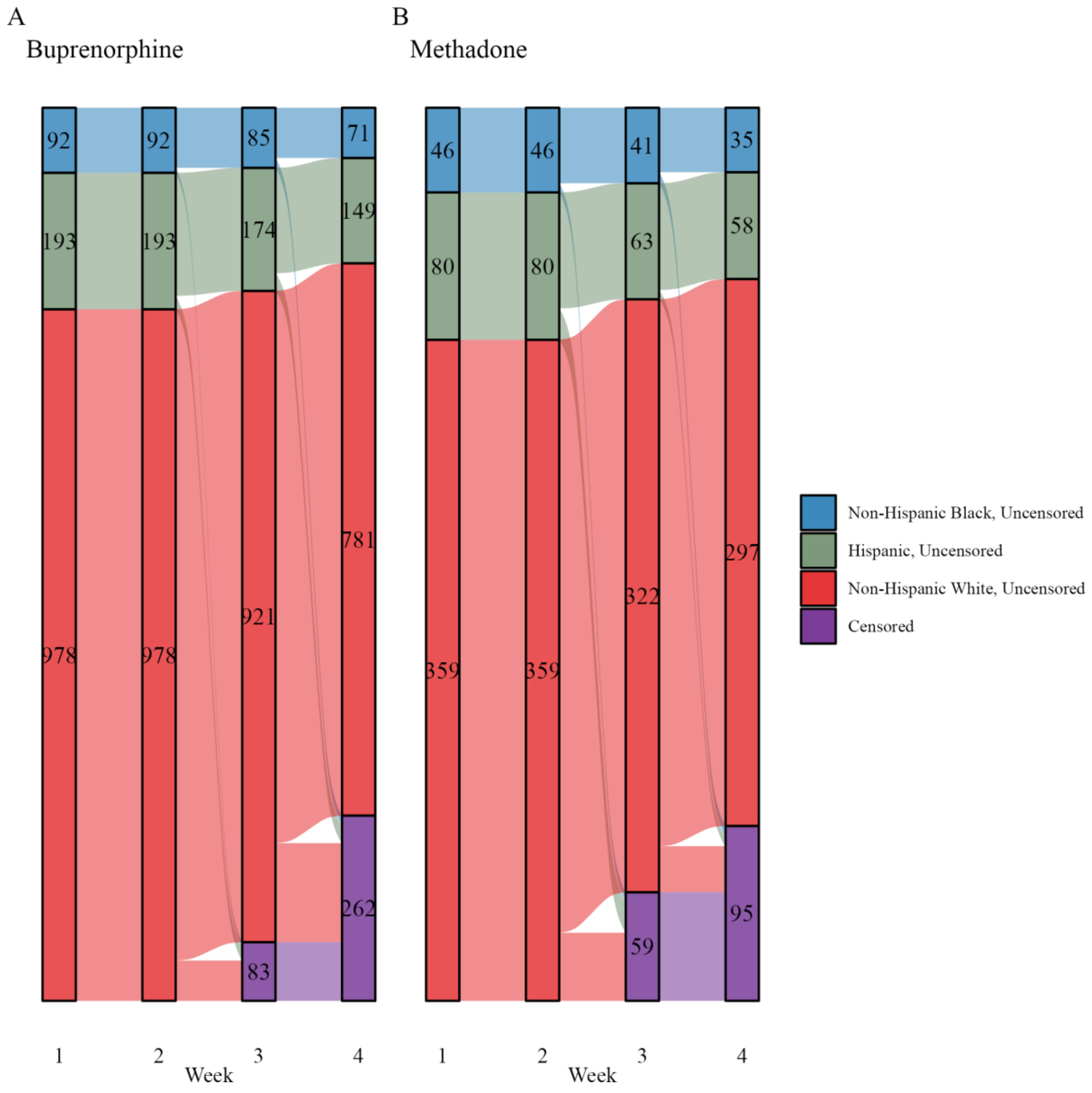

© 2024 Ross RK et al. *JAMA Network Open*.

eFigure 2. Violin Plots of Maximum Daily Dose of Buprenorphine Stratified by Self-Classified Race and Ethnicity for Each Trial

The black horizontal line in each violin marks the mean for that race-ethnicity group.

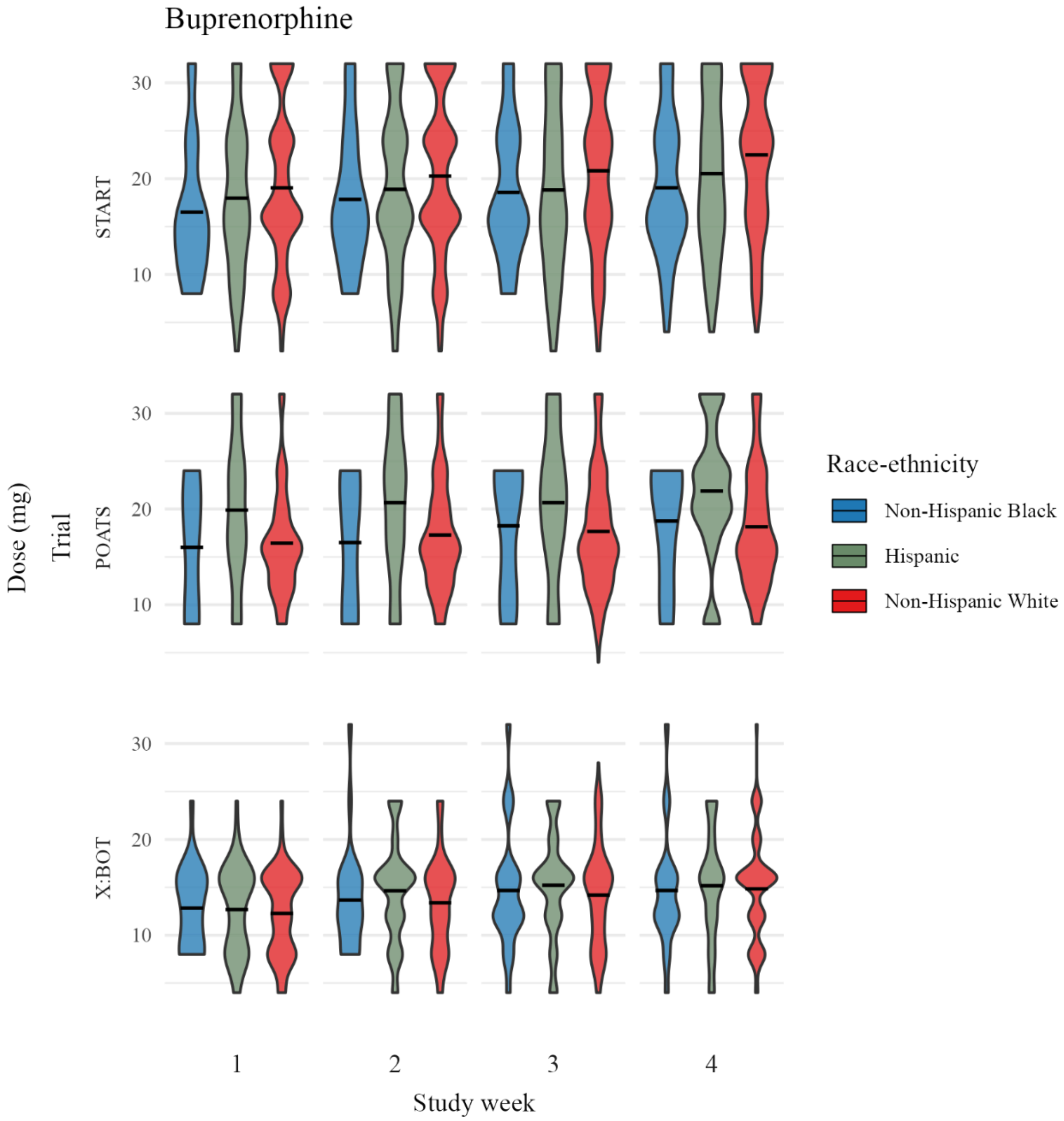

© 2024 Ross RK et al. *JAMA Network Open*.

eTables

eTable 1. Description of Patient Sample Included in Buprenorphine Analysis, Stratified by Trial

	Non-Hispanic White	Non-Hispanic Black	Hispanic
		**START**	
N	483	60	122
Sex			
Male	339	41	81
Female	144	19	41
Age'	32 (26-44)	49 (43-55)	40 (33-47)
		**POATS**	
N	319	8	18
Sex			
Male	183	5	11
Female	136	3	7
Age'	29 (25-38)	31 (29-47)	29 (25-42)
		**X-BOT**	
N	176	24	53
Sex			
Male	117	22	42
Female	59	2	11
Age'	31 (26-38)	45 (31, 48)	30 (26, 39)

' Median (interquartile range)

© 2024 Ross RK et al. *JAMA Network Open*.

eTable 2. Analyses Stratifying or Standardizing by Trial: Maximum Daily Dose of Buprenorphine in Week 4 of Treatment, Standardized by Age and Sex

	Non-Hispanic White	Non-Hispanic Black		Hispanic	
	**Mean**	**Mean**	**Difference**[1]	**Mean**	**Difference**[1]
**Stratified by trial**					
START	22.6 (21.8, 23.4)	19.7 (17.5, 21.9)	-2.9 (-5.3, -0.5)	20.4 (18.4, 22.4)	-2.2 (-4.4, 0.0)
POATS	18.2 (17.5, 18.9)	19.1 (11.3, 26.8)	0.9 (-6.9, 8.7)	21.9 (18.1, 25.7)	3.7 (-0.2, 7.6)
X-BOT	14.8 (14.0, 15.7)	13.6 (11.1, 16.0)	-1.3 (-3.8, 1.3)	15.0 (13.3, 16.7)	0.2 (-1.7, 2.1)
**Standardized by trial**					
All 3 trials	19.8 (19.3, 20.3)	18.2 (16.2, 20.2)	-1.6 (-3.7, 0.5)	19.9 (18.4, 21.4)	0.1 (-1.5, 1.7)
POATS excluded	20.5 (19.8, 21.2)	18.0 (16.2, 19.8)	-2.5 (-4.3, -0.6)	19.1 (17.7, 20.4)	-1.4 (-2.9, 0.1)

[1] Difference compared to non-Hispanic White patients

© 2024 Ross RK et al. *JAMA Network Open*.

eTable 3. Mean Methadone Dose From Sensitivity Analysis in Which Methadone Outlier Doses Were Replaced, Standardized by Age and Sex

	Non-Hispanic White	Non-Hispanic Black		Hispanic	
**Standardized by age + sex**					
	**Mean**	**Mean**	**Difference**[1]	**Mean**	**Difference**[1]
Week 3	67.9 (65.4, 70.3)	52.6 (42.1, 63.1)	-15.3 (-26.0, -4.5)	64.9 (59.5, 70.3)	-3.0 (-8.9, 3.0)
Week 4	72.9 (70.2, 75.7)	56.5 (43.0, 69.9)	-16.5 (-30.2, -2.7)	68.7 (62.5, 74.8)	-4.3 (-11.0, 2.5)
**Standardized by full covariates set**					
	**Mean**	**Mean**	**Difference**[1]	**Mean**	**Difference**[1]
Week 3	67.7 (65.3, 70.2)	55.4 (41.7, 69.0)	-12.4 (-26.3, 1.5)	63.9 (56.6, 71.1)	-3.9 (-11.5, 3.8)
Week 4	72.6 (69.8, 75.3)	59.0 (48.1, 69.9)	-13.6 (-24.8, -2.3)	68.8 (62.0, 75.6)	-3.8 (-11.2, 3.6)

[1] Difference compared to White patients

© 2024 Ross RK et al. *JAMA Network Open*.

eTable 4. Analysis Stratified by Severity of Baseline Withdrawal Symptoms: Differences in Maximum Daily Dose and Percentage Receiving a Higher Dose in Week 4 of Treatment, Standardized by Age and Sex

	**Black-White difference**		**Hispanic-White difference**	
	**Moderate/severe**	**Mild/none**	**Moderate/severe**	**Mild/none**
Buprenorphine dose (mg)[1]	-2.8 (-5.5, -0.2)	-2.5 (-5.5, 0.6)	0.7 (-1.2, 2.5)	-0.8 (-2.8, 1.3)
Methadone dose (mg)[2]	-10.4 (-18.7, -2.2)	-14.4 (-27.3, -1.4)	-8.4 (-19.8, 3.0)	-1.7 (-10.1, 6.7)
% receiving a higher dose[3,4]	-11.2 (-33.2, 10.8)	-17.3 (-34.8, 0.1)	2.4 (-7.5, 12.3)	-5.8 (-15.4, 3.8)

[1] Moderate/severe: non-Hispanic Black N = 27, Hispanic N = 83, non-Hispanic White N = 523; Mild/none: non-Hispanic Black N = 65, Hispanic N = 108, non-Hispanic White N = 451

[2] Moderate/severe: non-Hispanic Black N = 17, Hispanic N=39, non-Hispanic White N = 160; Mild/none: non-Hispanic Black N = 29, Hispanic N = 41, non-Hispanic White N = 196

[3] Higher dose defined as buprenorphine ≥16mg or methadone ≥60mg

[4] Moderate/severe: non-Hispanic Black N = 44, Hispanic N = 122, non-Hispanic White N = 683; Mild/none: non-Hispanic Black N = 94, Hispanic N = 149, non-Hispanic White N = 647

© 2024 Ross RK et al. *JAMA Network Open*.

eReferences.

[1] Van Der Laan Mark J, Rubin Daniel. Targeted maximum likelihood learning *The international journal of biostatistics.* 2006;2.

[2] Williams Nicholas, D´ıaz Iván. lmtp: An R package for estimating the causal effects of modified treatment policies *Observational Studies.* 2023.

[3] D´ıaz Iván, Williams Nicholas, Hoffman Katherine, Schneck Edward. Non-parametric causal effects based on longitudinal modified treatment policies *Journal of the American Statistical Association.* 2021.

[4] Laan Mark J., Polley Eric C., Hubbard Alan E.. Super Learner *Statistical Applications in Genetics and Molecular Biology.* 2007;6.

[5] Polley Eric C., Laan Mark J.. Super Learner In Prediction Working Paper 266.U.C. Berkeley Division of Biostatistics Working Paper Series 2010.

[6] Tibshirani Robert. Regression shrinkage and selection via the lasso *Journal of the Royal Statistical Society Series B: Statistical Methodology.* 1996;58:267–288.

[7] Friedman Jerome H. Multivariate adaptive regression splines *The annals of statistics.* 1991;19:1–67.

[8] Wright Marvin N., Ziegler Andreas. ranger: A Fast Implementation of Random Forests for High Dimensional Data in C++ and R *Journal of Statistical Software.* 2017;77:1–17.

[9] Chen Tianqi, Guestrin Carlos. Xgboost: A scalable tree boosting system in *Proceedings of the 22nd acm sigkdd international conference on knowledge discovery and data mining*:785–794 2016.

[10] Phillips Rachael V, Laan Mark J, Lee Hana, Gruber Susan. Practical considerations for specifying a super learner *International Journal of Epidemiology.* 2023;52:1276–1285.

© 2024 Ross RK et al. *JAMA Network Open*.
