# Supplementary material for: Variation in Opioid Agonist Dosing in Clinical Trials by Race and Ethnicity
Source: JAMA Netw Open. 2024 Oct 4;7(10):e2436612. doi: 10.1001/jamanetworkopen.2024.36612 (PMC11581645; doi:10.1001/jamanetworkopen.2024.36612)
Supplement: Supplement 2. — Data Sharing Statement [file jamanetwopen-e2436612-s002.pdf]

Data Sharing Statement

Ross. Variation in Opioid Agonist Dosing in Clinical Trials by Race and Ethnicity. *JAMA Netw Open*. Published October 04, 2024. doi:10.1001/jamanetworkopen.2024.36612

Data
Data available: Yes
Data types: Deidentified participant data
How to access data: A version of the deidentified data are publicly available: https://github.com/CTN-0094/public.ctn0094data. For our analysis, we used a version of the data provided directly to us by the investigators who conducted the data harmonization (under CTN-0094). R code for analysis is available: https://github.com/CI-NYC/race_dose
When available: beginning date: 09-21-2023

Supporting Documents
Document types: Statistical/analytic code
How to access documents: A version of the deidentified data are publicly available: https://github.com/CTN-0094/public.ctn0094data. For our analysis, we used a version of the data provided directly to us by the investigators who conducted the data harmonization (under CTN-0094). R code for analysis is available: https://github.com/CI-NYC/race_dose
When available: With publication

Additional Information
Who can access the data: The data are publicly available
Types of analyses: Any
Mechanisms of data availability: They are available at the url provided above
